# Dysfunction of Nrf-2 in CF Epithelia Leads to Excess Intracellular H_2_O_2_ and Inflammatory Cytokine Production

**DOI:** 10.1371/journal.pone.0003367

**Published:** 2008-10-10

**Authors:** Junnan Chen, Michael Kinter, Samuel Shank, Calvin Cotton, Thomas J. Kelley, Assem G. Ziady

**Affiliations:** 1 Department of Pediatrics, Case Western Reserve University, Cleveland, Ohio, United States of America; 2 Free Radical Biology and Aging Research Program, Oklahoma Medical Research Foundation, Oklahoma City, Oklahoma, United States of America; University of Giessen Lung Center, Germany

## Abstract

Cystic fibrosis is characterized by recurring pulmonary exacerbations that lead to the deterioration of lung function and eventual lung failure. Excessive inflammatory responses by airway epithelia have been linked to the overproduction of the inflammatory cytokine IL-6 and IL-8. The mechanism by which this occurs is not fully understood, but normal IL-1β mediated activation of the production of these cytokines occurs via H_2_O_2_ dependent signaling. Therefore, we speculated that CFTR dysfunction causes alterations in the regulation of steady state H_2_O_2_. We found significantly elevated levels of H_2_O_2_ in three cultured epithelial cell models of CF, one primary and two immortalized. Increases in H_2_O_2_ heavily contributed to the excessive IL-6 and IL-8 production in CF epithelia. Proteomic analysis of three in vitro and two in vivo models revealed a decrease in antioxidant proteins that regulate H_2_O_2_ processing, by ≥2 fold in CF vs. matched normal controls. When cells are stimulated, differential expression in CF versus normal is enhanced; corresponding to an increase in H_2_O_2_ mediated production of IL-6 and IL-8. The cause of this redox imbalance is a decrease by ∼70% in CF cells versus normal in the expression and activity of the transcription factor Nrf-2. Inhibition of CFTR function in normal cells produced this phenotype, while N-acetyl cysteine, selenium, an activator of Nrf-2, and the overexpression of Nrf-2 all normalized H_2_O_2_ processing and decreased IL-6 and IL-8 to normal levels, in CF cells. We conclude that a paradoxical decrease in Nrf-2 driven antioxidant responses in CF epithelia results in an increase in steady state H_2_O_2_, which in turn contributes to the overproduction of the pro-inflammatory cytokines IL-6 and IL-8. Treatment with antioxidants can ameliorate exaggerated cytokine production without affecting normal responses.

## Introduction

Cystic Fibrosis (CF) is an autosomal recessive genetic disorder caused by a genetic defect in the cystic fibrosis transmembrane conductance regulator (CFTR), a protein that functions primarily as a chloride channel [1]. The most common mutation in humans (ΔF508) results in the misprocessing, subsequent degradation, and loss of function of CFTR [1]. This results in the dysregulation of ion and fluid transport across the epithelium and a number of secondary defects that exacerbate inflammation, which in the airways culminate in respiratory failure [2].

A hall mark of CF lung disease is exaggerated production of inflammatory cytokines, such as IL-6 [3] and IL-8 [4], which result in excessive inflammation. Shortly after birth, early onset of lung infection and the accompanying inflammatory response become self sustaining [5], and ultimately destroy the airways, impair gas exchange, and lead to respiratory failure and death. Epithelial cells, a primary site of dysfunction in CF, are major contributors to the inflammatory cascades involved in disease. Anti-inflammatory therapy is effective in limiting lung deterioration [5], but adverse effects have discouraged the use of both steroidal and non-steroidal drugs. Nevertheless, controlling inflammation appears to slow disease progression.

The series of events that link CFTR dysfunction to inflammation are not well understood, but may well be a key to controlling lung disease in CF, and may be a good site for therapeutic intervention. A potential mechanism for the perpetual production of inflammatory cytokines observed in CF is oxidative stress, which results from an imbalance of oxidants and anti-oxidants in the cell [6]–[9]. As the chief oxidant in cells is H_2_O_2_, recent reports that IL-1β signaling in epithelial cells is mediated by H_2_O_2_ [10] support the notion that oxidant imbalances in CF cells would contribute to exaggerated inflammatory responses. Since epithelial cells are central to inflammatory pathways in the lung [1]–[5], it is logical to examine the redox potential of CF epithelia. To date only one study, utilizing fluorescent indicators, has reported that no differences in intracellular redox potential are observed between CF and corrected cells [11]. However, no analysis of intracellular steady-state H_2_O_2_ concentration in CF epithelia has been conducted.

Delineating mechanisms of pulmonary inflammation in CF is perhaps the most pressing need in the field [2], [5]. Therefore, we sought to test the hypothesis that excessive inflammation in CF is triggered by the accumulation of intracellular H_2_O_2_. To increase confidence in our results we studied five different models of CF epithelia, three *in vitro* and two *in vivo*. Our data reveal a significant increase in H_2_O_2_ in the presence and absence of inflammatory stimuli in the CF state. This increase heavily contributes to the production of the inflammatory cytokine, IL-6 and IL-8.

To investigate the mechanism of H_2_O_2_ elevation, we used 2D electrophoresis and tandem mass spectrometry to examine the expression of redox proteins that regulate intracellular peroxide. We found significant decreases in the expression of a number of antioxidant enzymes, including thioredoxin-1 (TRX-1), peroxiredoxin-1 (PRDX-1), peroxiredoxin-6 (PRDX-6), catalase, and glutathione-S-transferase-pi (GST-pi), but a marked increase in Mn superoxide dismutase (SOD2). This pattern of protein expression is consistent with an environment in which H_2_O_2_ production is increased by the SOD2 but the metabolism of H_2_O_2_ by catalase, PRDX-1, and PRDX-6 is reduced, allowing a net accumulation of intracellular peroxide.

The observation of changes in the expression of antioxidant enzymes led to an evaluation of the antioxidant response element (ARE). We found that the expression and activity of the transcription factor that facilitates ARE responses, Nrf-2 [12], was decreased by approximately 70% in CF cells vs. normal. Importantly, we were able to rescue CF cells by treatment with antioxidants N-acetyl cysteine or selenium, stabilization of Nrf-2, or the overexpression of Nrf-2. These approaches all normalized H_2_O_2_ processing and decreased IL-6 and IL-8 cytokine production to normal levels in CF cells.

Taken together, the data present novel evidence that oxidative stress in CF epithelia is not resolved through the ARE due to the dysfunction of Nrf-2. Coupled with an increase in SOD2, loss of Nrf-2 function in CF cells leads to excess intracellular H_2_O_2_, which mediates exaggerated inflammatory cytokine production in CF epithelia. In the context of disease, this additional component of inflammation in CF is a good target for therapeutic intervention, and our data suggest that treatments that counter elevations in H_2_O_2_ can alleviate the CF hyper inflammatory phenotype.

## Results

### Intracellular hydrogen peroxide levels are elevated in CF airway epithelia

**Figure 1:**
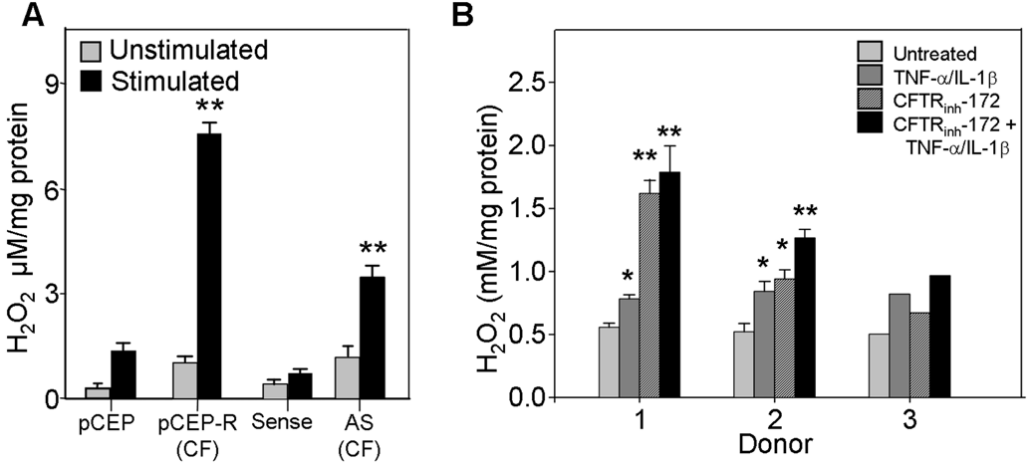
Steady state hydrogen peroxide levels are elevated in CF in the absence and presence of inflammatory stimulation. H_2_O_2_ levels were assayed in: A) Two immortalized cell line pair models of CF, the 16 HBEo− (sense and antisense (AS)) and 9 HTEo− (pCEP and pCEP-R), and; B) one polarized primary cell model, the wd-HPTE (untreated or treated with 20 µM CFTR_inh_-172 for 72 hrs.). Unstimulated cells are compared to cells harvested 24 hrs following 1 hr. incubation with TNF-α/IL-1β (10 ng/ml each). * connotes significant difference from unstimulated normal control (p<0.05), while ** connotes significant difference from both unstimulated and stimulated normal control. Each data bar represents the average of 8 replicate wells in 4 experiments for (A), or 3 replicate wells from donors 1 and 2, or 2 replicate wells for donor 3 in 1 experiment for (B).

In initial studies we compared the cultured CF model cell pairs 9HTEo^−^ pCEP (normal) and pCEP-R (CF), and the 16HBEo^−^ S (normal) and AS (CF). We examined H_2_O_2_ levels in CF versus normal cells. In immortalized cell lines, unstimulated CF cells produce significantly higher levels of H_2_O_2_ compared with normal matched pairs (Figure 1a). These levels are further increased 24 hrs following stimulation with a cocktail of the inflammatory cytokines TNF-α/IL-1β (Figure 1a). Our measurements reflect steady state H_2_O_2_ in the cells. Since significant increases were observed in the 9HTEo^−^ pCEP-R which express CFTR but lack its function, we tested the effect of the inhibition of CFTR on steady state H_2_O_2_ levels in well differentiated human primary tracheal epithelia (wdHPTE) from three different donors. Levels of peroxide significantly increased following pharmacological inhibition of CFTR for 72 hrs with 20 µM CFTR_inh_-172 (Figure 1b), compared to “same donor” cells that were not treated with inhibitor. Stimulation with TNF-α/IL-1β further increased H_2_O_2_ levels (Figure 1b). These results were reproduced in cells from all three donors.

In all, these studies demonstrate that CF cells exhibit an increase in intracellular H_2_O_2_ in the basal state that is enhanced by TNF-α/IL-1β stimulation.

### H_2_O_2_ increases the production of IL-6 and IL-8

To examine whether the elevations in H_2_O_2_ levels that we observe contribute to the exaggerated inflammatory response in CF, we tested the effect of H_2_O_2_, N-acetyl cysteine (NAC), or selenium (Se) on cytokine production by our normal and CF immortalized cell pairs. For these studies all cells were stimulated with TNF-α and IL-1β. The addition of H_2_O_2_ to the media of the cells increases [9], while the addition of NAC or Se decreases intracellular H_2_O_2_ [13].

**Figure 2:**
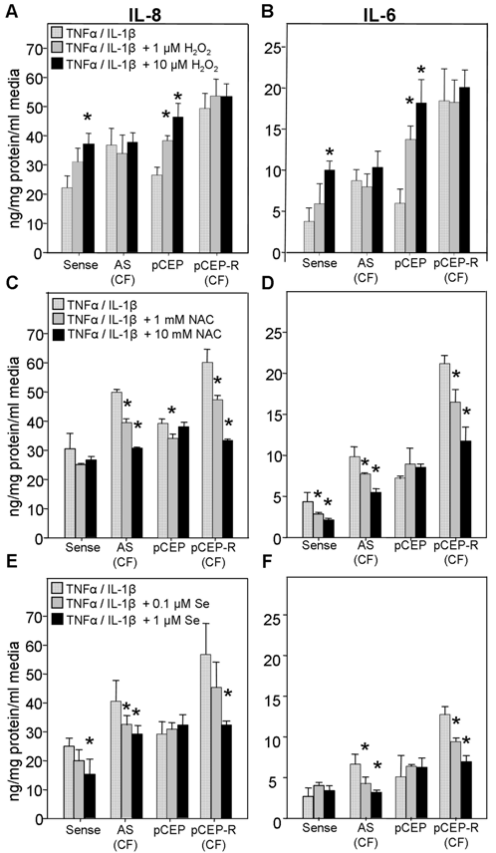
H_2_O_2_ and antioxidants significantly modulate the production of IL-6 and IL-8 in cells stimulated with TNFα/IL-1β. Normal and CF matched pair cell lines were stimulated for 1 hr. with TNFα/IL-1β (10 ng/ml each) and then cultured for 24 hrs. in the absence, or presence of 1 µM or 10 µM of H_2_O_2_, 1 mM or 10 mM *N*-acetyl cysteine (NAC), or 0.1 µM or 1 µM selenium (Se). * connotes significant difference (p<0.05) from untreated control (spotted white bars). Each data bar represents the average of 8 replicate wells in 3 experiments for A–F.

Both TNF-α/IL-1β stimulated 9HTEo^−^ and 16HBEo^−^ normal cell line pairs exhibited significant elevations in IL-8 and IL-6 production following addition of H_2_O_2_ (1 or 10 µM), while no significant increase was observed in corresponding CF cell pairs (Figure 2a, b). This is consistent with the notion that peroxide mediated cytokine production in CF cells is already present at maximal levels. Conversely when TNF-α/IL-1β stimulated cells were treated with NAC (1 or 10 mM), a known modulator of H_2_O_2_ [13], IL-6 and IL-8 production was significantly decreased, especially in the CF cell pairs (Figure 2c, d). In these cells, where cytokine secretion is on average 1.5 fold higher than normal matched pairs following inflammatory stimulation, NAC decreased responses to the normal levels exhibited by stimulated normal cells. Finally, we tested the effect of Se, which enhances the activity of seleno-proteins such as TRX-1 and PRDX-1 and 6. A significant decrease in cytokine production was also observed 24 hrs after we supplemented our media with Se (0.1 or 1.0 µM), (Figure 2e, f). While antioxidant treatment consistently decreased cytokine responses in CF cells, the effect in normal cells was not as robust. This is consistent with the notion that antioxidant mechanisms in CF cells are more compromised versus normal cells, and therefore a more beneficial effect of antioxidants is observed in those cells. Responses to H_2_O_2_, NAC, and Se were dose dependent.

### Differential expression of proteins that regulate H_2_O_2_ in CF airway epithelia

**Figure 3:**
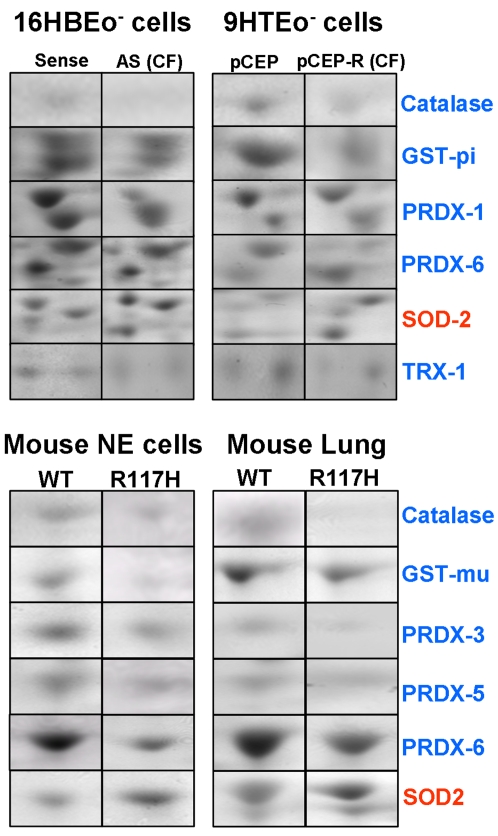
Expression of antioxidant proteins is altered in cystic fibrosis. Non-stimulated models of CF are compared by 2-D gel analysis. For *in vitro* studies, 16HBEo^−^ (sense and antisense (AS)) and 9HTEo^−^ (pCEP and pCEP-R) cell line pairs are grown to 80% confluence on 5 different occasions, homogenized, and the homogenates equally loaded onto 2-D gels. For *in vivo* studies, 5 sets of excised nasal epithelial or whole lung tissues from 8 week old wildtype mice or their CF litter mates were harvested, homogenized and total protein analyzed. Gels were scanned by densitometer, averaged and a comparative densities calculated (see Tables 1 and 2). Representative 2-D gel bands from CF and normal matched pairs are shown for each model. Blue protein names indicate a decrease, while red names indicate an increase in expression in CF.

To investigate the mechanism of the increase of H_2_O_2_ in CF cells, we used a proteomic approach to analyze relevant redox proteins. We examined 2-D gels equally loaded with whole cell protein from the cultured CF model 9HTEo^−^ and 16HBEo^−^ cell pairs. Comparisons of five gel maps for untreated normal and CF cells indicated the differential expression by 2 fold or higher of the key redox proteins thioredoxin 1 (TRX-1), Mn superoxide dismutase (SOD2), glutathione-S-transferase pi (GST-pi), peroxiredoxin (PRDX) 6, TRX dependent peroxide reductase (PRDX-1), and catalase (Figure 3, Table 1). Spots were identified using LC-MS to obtain CID spectra with sequence information (example CID spectra, Figure S1a–f), that were each matched to the human protein database (NCBI) with greater than 95% confidence (Table S1). Our protein identifications are based on a sequence coverage range average of 28%–67% (Table S1). This translates to 4–24 different tryptic peptides whose sequence match entries in public databases for each protein, further increasing our confidence in our identifications. Importantly, for each of our bands of interest we only detected tryptic peptides belonging to an individual protein. The lack of detection of any peptides from secondary proteins indicated that the level of any contamination (>10 fold less abundant) would not affect our accuracy of quantitation, and that the direct comparison of Coomassie stained 2D gel bands for this set of proteins was valid.

**Table 1:** Differential expression of redox regulating proteins in cultured CF epithelia.

	Average fold change in 9HTEo^−^ pCEP-R		Average fold change in 16HBEo^−^ AS	
Protein name	Non-stimulated	Stimulated	Non-stimulated	Stimulated
**Catalase**	**D** 2.82±0.2	**D** 3.35±0.12	**D** 2.55±0.28	**D** 2.87±0.11
**GST-pi**	**D** 4.86±0.25	**D** 6.20±0.25	**D** 2.83±0.30	**D** 2.50±0.21
**PRDX-1**	**D** 2.18±0.17	**D** 2.40±0.31	**D** 2.67±0.16	**D** 3.28±0.25
**PRDX-6**	**D** 2.84±0.33	**D** 4.84±0.23	**D** 3.50±0.22	**D** 4.12±0.20
**SOD2**	**U** 4.37±0.43	**U** 2.35±0.35	**U** 4.25±0.15	**U** 2.12±0.23
**TRX-1**	**D** 2.35±0.21	**D** 3.32±0.39	**D** 2.25±0.22	**D** 2.67±0.24

Comparison of 2D gels revealed that in the absence of inflammatory stimulus CF cells exhibit a marked decrease in the expression of TRX-1, PRDX-1 and 6, catalase and GST-pi, but a marked increase in SOD2 compared with normal matched cell pairs (Table 1). Assays of total cell SOD (Figure S2), revealed a significant increase in activity in CF epithelia, consistent with our proteomic data and an increase in SOD2 expression. These significant differences were present in the 9HTEo^−^ and 16HBEo^−^ cell line pairs, although their extent varied (Table 1). When we exposed cells to TNFα/IL-1β, differences between CF and normal cell lines increased for TRX-1, PRDX-1 and 6, and GST-pi, but diminished for catalase and SOD2 (Table 1). These changes suggest that accumulation of H_2_O_2_ following inflammatory stimulus might be predominantly due to decreases in peroxidase activity rather than further increases in H_2_O_2_ production.

To test whether differences in the redox proteins observed in CF cells extended to an *in vivo* model of CF, we examined protein expression, by 2-D gel analysis, in the excised nasal epithelia (NE) and whole lungs of R117H mutant mice compared with normal littermates. While epithelial cells are the predominant cell type in excised NE, they present a smaller contribution in whole lung. We found decreases in catalase, GST-mu, PRDX-3, 5 and 6, and an increase in SOD2 (Figure 3, Table 2) in both comparisons of NE and whole lung. No significant difference was found in the expression of PRDX-1 or TRX-1. Nevertheless, the pattern of protein expression in the CF mouse model mirrored the pattern observed *in vitro*, where enzymes that produced H_2_O_2_ are increased while ones that metabolize it are reduced. Identifications were based on sequence coverage that ranged from 23–58%, and 7–26 tryptic peptides sequenced for each of the proteins (example CID spectra, Figure S3a–g).

**Table 2:** Differential expression of redox regulating proteins in CF mouse nasal epithelia and whole lung.

Protein	Fold change in CF NE	Fold change in CF lungs	Average sequence coverage (%)	Average number of peptide ions	ID probability (*p* value)
**Catalase**	**D** 2.22±0.15	**D** 3.25±0.30	36±4	26±3	<0.001
**GST-mu**	**D** 7.42±0.51	**D** 2.34±0.15	57±3	23±2	<0.000001
**PRDX-3**	**D** 3.82±0.38	**D** 2.12±0.22	23±2	7±2	<0.001
**PRDX-5**	**D** 4.67±0.60	**D** 3.45±0.20	26±4	11±2	<0.001
**PRDX-6**	**D** 5.72±0.68	**D** 2.05±0.16	58±6	22±5	<0.000001
**SOD2**	**U** 5.23±0.55	**U** 3.84±0.34	34±7	12±3	<0.0001

### Nrf-2 is dysfunctional CF airway epithelia

**Figure 4:**
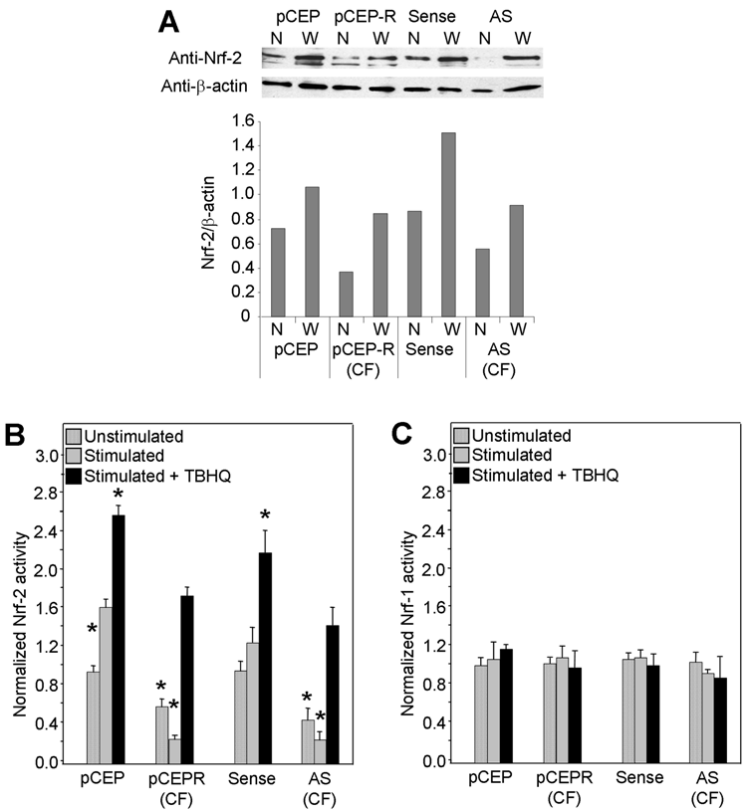
Nrf-2 expression and activity are decreased in CF epithelia in the absence and presence of inflammatory stimulation. Normal and CF matched cell pairs are co-transfected with either a plasmid coding for Firefly luciferase expression driven by a Nrf-2 or Nrf-1 promoter and one coding for Renilla luciferase driven by the CMV promoter. Panel A: Western analysis of nuclear (N) or whole cell (W) Nrf-2 and an anti-β actin control. Band densities are used to calculate the relative abundance chart. Panel B: Cells transfected with the Nrf-2 promoter luciferase construct. Panel C: Cells transfected with the Nrf-1 promoter luciferase construct. Two days following transfection cell homogenates were assayed for luciferase activity by luminometer. Unstimulated cells are compared to cells stimulated with TNFα/IL-1β (10 ng/ml each) alone, or in the presence of an activator of Nrf-2 but not Nrf-1, tBHQ. * connotes significant difference (p<0.05) from respective normal control stimulated with TNFα/IL-1β (10 ng/ml each) alone. Each data bar represents the average of 8 replicate wells in 3 experiments for B and C.

The differential expression of antioxidant proteins the regulate H_2_O_2_ indicates that ARE responses are deficient in CF epithelia. Therefore, we next tested the activity of the central ARE transcription factor Nrf-2. Western analysis revealed that levels of Nrf-2 protein are decreased in both the cytoplasm and nucleus of the CF cell pairs (Figure 4a). For the 9HTEo^−^ cell pairs, CF cell levels of Nrf-2 were decreased by 49.8% in nuclear, and 21.2% in whole cell extracts. For the 16HBEo^−^ cells pairs, CF cell levels of Nrf-2 were decreased by 35.2% in nuclear, and 39.3% in whole cell extracts.

We then tested the activity of Nrf-2 using a luciferase expression plasmid driven by a Nrf-2 promoter. Cells were co-transfected with a Renilla luciferase construct to control for transfection efficiency. CF cells exhibited normalized expression levels ∼55–60% lower than normal matched pairs (Figure 4b). Stimulation with TNFα/IL-1β resulted in a further decrease in CF cell luciferase expression versus an increase in normal controls, indicating a drop in Nrf-2 activity of 75–85% in CF cells (Figure 4b). In our first attempt to rescue the CF phenotype we treated our cell models with *tert*-Butylhydroquinone (tBHQ), an activator of Nrf-2 by stabilization against degradation [14]. Treatment reversed decreased Nrf-2 activity and significantly increased luciferase expression in CF cell lines stimulated with TNF-α/IL-1β (Figure 4b). Importantly, this increase of Nrf-2 activity in stimulated tBHQ treated CF cells is comparable to levels observed in stimulated normal cells, indicating a fundamental correction of the defect.

To insure that the luciferase expression that we observe reflects Nrf-2 promoter activity in the epithelial cell lines and not a transfection artifact, we tested luciferase expression driven by the Nrf-1 promoter. We used a construct identical to the Nrf-2 construct with the exception of promoter region, which binds Nrf-1. No significant difference in Nrf-1 activity was observed between CF and normal cell lines in the absence or presence of inflammatory stimulus (Figure 4c). These control data demonstrate that our test of Nrf-2 activity is specific and not simply a product the effect of DNA transfection in our cell lines.

### CFTR dysfunction reduces Nrf-2 activity and increases intracellular H_2_O_2_

**Figure 5:**
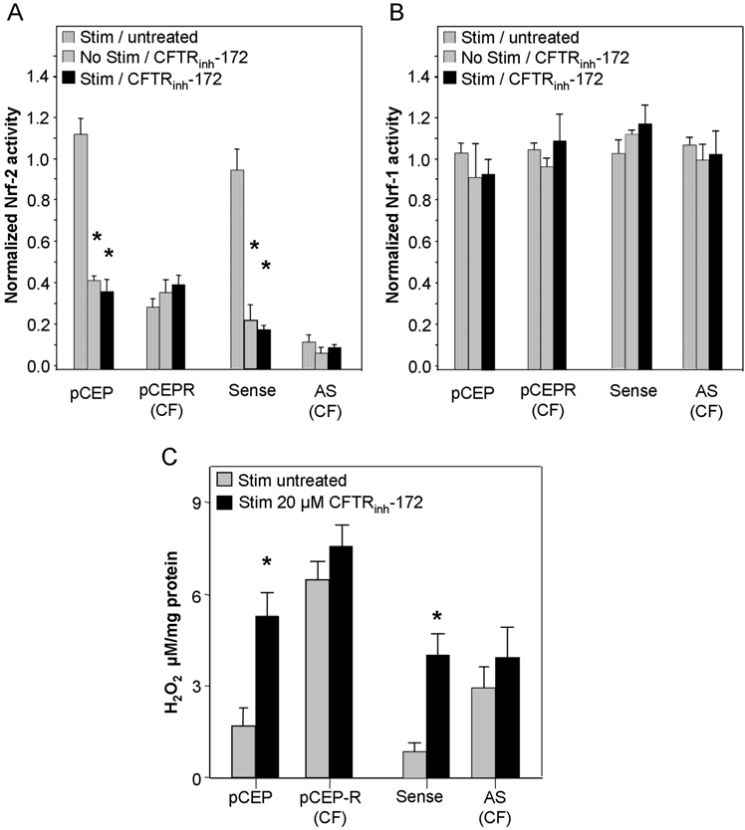
Inhibition of CFTR activity decreases Nrf-2 activity and increases H_2_O_2_ levels. Normal and CF matched cell pairs incubated with 20 µM CFTR_inh_-172 are co-transfected with either a plasmid coding for Firefly luciferase driven by a Nrf-2 (Panel A) or Nrf-1 (Panel B) promoter, and one coding for Renilla luciferase driven by the CMV promoter. Three days following transfection, normalized luciferase activity is measured. Panel C: H_2_O_2_ levels in cells incubated with CFTR_inh_-172 for 72 hrs and stimulated with TNFα/IL-1β (10 ng/ml each). * connotes significant difference (p<0.05) from respective uninhibited controls. Each data bar represents the average of 8 replicate wells in 3 experiments for A, B, and C.

Since the loss of CFTR function (9HTEo^−^ cell pair, and CFTR inhibited wdHPTE) or expression (16HBEo^−^ cell pair) is the defining difference in our CF cell pairs, inhibiting CFTR in the normal cell pairs should produce the aberrations in H_2_O_2_ processing and Nrf-2 activity we observed in previous experiments. Therefore, to test this hypothesis, we used a pharmacological agent, CFTR_inh_-172, to inhibit CFTR activity in normal cell line controls. In CF cells, inhibition of CFTR for 72 hrs did not affect Nrf-2 activity (Figure 5a). In normal cells however, inhibition of CFTR significantly decreased Nrf-2 activity vs. non-inhibited control, by ∼80% in the 16HBEo^−^ and ∼70% in the 9HTEo^−^ cells, in the presence or absence of inflammatory stimulation. These findings were Nrf-2 specific as no significant differences were observed for Nrf-1 activity (Figure 5b). Decreases in Nrf-2 activity in CFTR inhibited normal cells correlates with a significant 3- to 4-fold increase in H_2_O_2_ versus non-inhibited controls (Figure 5c). No significant increase is observed in the CF cells lines, consistent with an already existing lack of CFTR function in these cells.

Therefore, in both tested normal models CFTR inhibition specifically decreases Nrf-2 activity and elevates H_2_O_2_ to CF levels, while no significant changes are observed in the CF cell pairs. Taken together, these data link CFTR function to Nrf-2 activity and changes in intracellular H_2_O_2_. This is an important validation of our cell line model systems.

### Increasing Nrf-2 activity decreases H_2_O_2_ levels and pro-inflammatory cytokine production

**Figure 6:**
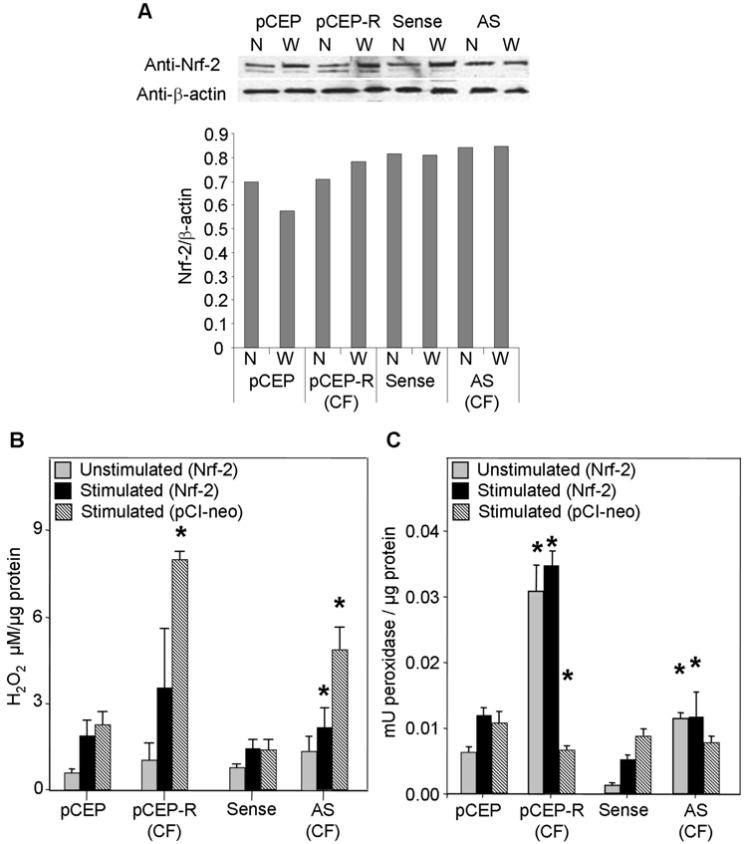
Overexpression of Nrf-2 reduces H_2_O_2_ and increases total peroxidase levels. 16HBEo^−^ and 9HTEo^−^ cell line pairs are co-transfected with either a mammalian expression plasmid for human Nrf-2 or the pCI-neo empty vector at 0.1 µg/5×10^5^ cells/well. H_2_O_2_ and total peroxidase levels are measured 48 hrs following transfection, and normalized to total cell protein concentration. Measurements of cell line pairs in the absence or presence of TNFα/IL-1β (10 ng/ml each) are shown. Panel A: Verification of Nrf-2 expression by Western blot in cells transfected with the Nrf-2 expression plasmid. Band densities are used to calculate relative abundance. Panel B: H_2_O_2_ levels normalized to protein concentration. Panel C: Total peroxidase units normalized to protein concentration. * connotes significant difference compared with respective normal control (Sense or pCEP cells). Each data bar represents the average of 6 replicate wells in 4 experiments for B and C.

Previous experiments suggest that a potential mechanism for the elevation of the inflammatory cytokines IL-6 and IL-8 in CF cells is an elevation of H_2_O_2_, which is mediated through the dysregulation of Nrf-2. To further validate this mechanist data, we tested the hypothesis that correcting Nrf-2 activity in CF cells would reverse the misprocessing of H_2_O_2_ and decrease excessive cytokine production. We used two approaches. First, we transfected CF cell pairs with an expression plasmid for Nrf-2 to increase expression to normal levels (Figure 6a). When the CF cells pairs over express Nrf-2, H_2_O_2_ decreases to levels not different from normal controls (Figure 6b), in contrast to non-transfected cell values (Figure 1). This decrease, in transfected CF cells pairs, is accompanied by a marked increase in peroxidase activity, consistent with an increase in antioxidant proteins expression (Figure 6c). No increase in Nrf-2 expression was observed in cells transfected with the pCI-neo vector (empty vector). Furthermore, transfection with an pCI-neo did not alter either peroxidase activity, or H_2_O_2_ levels in the CF cells.

**Figure 7:**
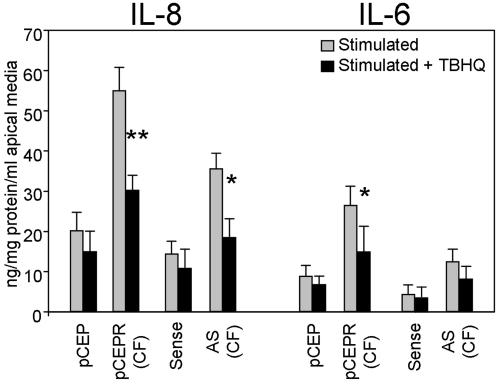
Stabilization of Nrf-2 reduces inflammatory cytokine production in stimulated epithelia. Levels of IL-8 or IL-6 secreted by normal and CF matched pairs following stimulation with TNFα/IL-1β (10 ng/ml each) alone, or TNFα/IL-1β (10 ng/ml each) in the presence of tBHQ. * connotes significant difference (p<0.05) from respective non-tBHQ treated control. Each data bar represents the average of 6 replicate wells in 3 experiments.

For our second strategy was to test the impact of improving Nrf-2 activity on inflammatory cytokine levels. We measured IL-6 and IL-8 levels in stimulated CF cells in the presence of tBHQ, which stabilizes Nrf-2 and increases its half life and activity (Figure 4b) [14]. Both IL-8 and IL-6 were decreased by as much 45% in stimulated CF cells cultured in the presence of tBHQ for 48 hrs compared with cells grown in its absence (Figure 7). The level of cytokine production exhibited by TNFα/IL-1β stimulated tBHQ treated CF cells was not significantly different from that observed for normal cell lines stimulated with TNFα/IL-1β. Taken together, the Nrf-2 expression and stabilization with tBHQ experiments demonstrate that correction of the reduced Nrf-2 activity in CF cells reduces H_2_O_2_ accumulation and inflammatory responses to normal levels. This is consistent with notion that dysregulation of Nrf-2 in CF epithelia is the key contributor to elevations of steady state H_2_O_2_ and the pro-inflammatory cytokines, IL-6 and IL-8.

## Discussion

CF is characterized by an exaggerated inflammatory response that becomes self sustaining and ultimately leads to the destruction of the lung [2]. Therefore, signaling mechanisms that increase inflammatory cytokine production are of great interest. Airway epithelia are a principle site of CFTR dysfunction in CF and a source of the excessive production of inflammatory cytokines, such as IL-6 and IL-8 [3], [4]. The mechanisms by which CF epithelia perpetuate the inflammatory cascades in the lung are not well understood, but H_2_O_2_ has recently been implicated in IL-1β mediated inflammatory signaling in these cells [10]. Furthermore, peroxide has been shown to stimulate inflammatory cytokine production [9], [15], [16]. We postulated that CFTR dysfunction may lead to increases in intracellular H_2_O_2_, and that this would increase production of IL-6 and IL-8 [9], [15], [16].

We found that steady state H_2_O_2_ is indeed increased in CF cell lines and primary epithelia following inhibition of CFTR channel function. The novel discovery of the elevation in intracellular H_2_O_2_ in CF epithelia, which we report here for the first time, coupled with strong evidence that this elevation significantly contributes to inflammatory responses, led to the investigation of the mechanism of H_2_O_2_ accumulation in CF cells. To do this we studied five different models of CF, using multiple approaches in multiple experiments to increase confidence in our findings.

First, we examined the source of increased H_2_O_2_. Based on experimental evidence, we suspect that this is the mitochondria. Our proteomic data indicate that the mitochondrial form of SOD, SOD2 exhibits elevated levels of expression in CF epithelia. This is further confirmed by biochemical data that shows significant increases in total cell SOD activity. Superoxide, which is converted to H_2_O_2_ by SOD2, is expected to be elevated in CF. Increased prenylation in CF epithelia has been shown to increase the activity of isoprenylated proteins such as Rho A [17] versus normal. Rac 1 activity, which is dependent on prenylation through the same pathway [18], would be increased leading to elevated NADPH oxidase (Nox 1) activity [19], increasing superoxide production. Coupled with increases in SOD2 expression and activity, increased H_2_O_2_ production, which we observe, would ensue.

Normally increases in oxidant load activate the ARE to produce proteins including TRX-1, and PRDX-1, 3, 5, and 6 [12]. In addition to catalase, these proteins make up the machinery that cells use to regulate H_2_O_2_. Therefore, to investigate the mechanism of increased intracellular H_2_O_2_, we examined the expression of the antioxidant proteins that regulate H_2_O_2_. We found that the expression of these proteins was significantly reduced in a number of *in vitro* and *in vivo* models of CF epithelia. To reduce the complexity of our *in vivo* samples, we used excised nasal tissue, which contains a high proportion of epithelial cells [20]. Differences in these cells agree with our data in vitro, and are even more pronounced than those observed in whole lungs. Nevertheless, whole lung protein exhibited differential expression similar to that observed in CF cultured epithelia, with increases in SOD2 and decreases in peroxidase enzymes. This may indicate that this phenomenon is systemic and not confined to epithelial cells. However, this remains to be tested.

The decrease in expression of protective proteins was linked to the dysfunction of Nrf-2. CF epithelia exhibited significantly lower expression and transcriptional activity of Nrf-2. When Nrf-2 was enhanced in CF cells by a number of approaches, intracellular H_2_O_2_ and cytokine production decreased to normal levels. Taken together our data strongly indicate that Nrf-2 dysfunction is present in CF epithelia and that, coupled with increased H_2_O_2_ production by SOD2, this results in the accumulation of H_2_O_2_.

Our data are supported by previous studies [21], [22]. Our observation for GST and PRDX agree with pervious reports on CF epithelial proteomes [21], [22]. Increases in steady state H_2_O_2_ levels are intriguing since this has been shown to be a potent activator of NF-κB [16], [23], and may be the chief mediator of IL-1β receptor signaling in CF epithelia [10]. An imbalance in the intracellular H_2_O_2_ would increase redox mediated activation of NF-κB, and contribute to the excessive production of IL-6 and IL-8, which is present in the CF lung and which we observe in our cultured cell models.

Lack of CFTR channel function, as is the case for our 9 HTEo^−^ pCEP-R or inhibited wd-HPTE cells, is sufficient for the elevation of H_2_O_2_. Inhibition of CFTR in normal cells produces elevation of H_2_O_2_ within 72 hrs. Previously reported [24] increases in cytokine production following CFTR inhibition are therefore driven, at least in part, by H_2_O_2_. Interestingly, CFTR may be the major transporter of GSH in epithelial cells [25], [26]. Disruption of the transport of GSH by the cell wide inhibition of CFTR may affect levels of GSH in the mitochondria, where it is transported to balance oxidant production. Furthermore, Nrf-2 activity is sensitive to GSH levels in the nucleus. If cellular levels of GSH are disrupted by the inhibition of CFTR, Nrf-2 activity may be affected. Therefore, the transport of GSH may be a CFTR function that is necessary for the normal regulation of the antioxidant response element in epithelial cells.

We observe a decrease in Nrf-2 levels both in the cytoplasm and nucleus in CF epithelia, compared to normal matched pairs. Three factors can contribute to this phenomenon, diminished transcription, accelerated degradation, and/or changes in binding partners. Transcription of Nrf-2 is sensitive to a number of regulatory mechanisms [13], including ones found to be dysfunctional in CF, such as lipid processing [20]–[22], [24], [27], and protein inhibitor of activated STAT3 activity [28]. The notion that correcting lipid processing would decrease inflammatory signaling is supported by reports that inhibitors of cholesterol synthesis, such as statins, act as anti NF-κB mediated-inflammatory agents in epithelial cells [29] and other tissues [30]. Furthermore, changes in energy consumption by the cell, which has been associated with CF [31], have been shown to impact Nrf-2 expression [32].

Ubiquitination and subsequent degradation of the Nrf-2 is modulated by Keap-1 [12], [33], whose interaction with Nrf-2 affects its rate of degradation and translocation to the nucleus. Increased oxidative stress results in the dissociation of Keap-1 from Nrf-2 in the cytoplasm [12], [33], exposing sites for phosphorylation, binding partner, as well as ubiquitination. Degradation of Nrf-2 will take place once it is released from Keap-1, if it remains in the cytoplasm and does not translocate to the nucleus. Determinants of nuclear translocation following dissociation from Keap-1 are not well understood, but may involve phosphorylation [33] and/or association with activating binding partners [12], [33]. If oxidative stress is elevated in CF, but Nrf-2 is not rapidly localized to the nucleus, a decrease in Nrf-2 levels due to ubiquitination and degradation would be expected, as we observe. This is consistent with our data for tBHQ, which stabilizes Nrf-2 [14]. CF cells treated with tBHQ exhibited increased Nrf-2 activity, as well as decreased IL-6 and IL-8 production. This suggests that differential degradation of Nrf-2 in CF epithelia plays a role in enhanced suppression of its activity.

Our data demonstrate that the ARE response pathway in CF cells is dysfunctional, with activation of Nrf-2 decreasing in the CF state both in the presence and absence of inflammatory stimulus. The down regulation of Nrf-2 in CF cells, although paradoxical considering the increased levels of H_2_O_2_, may be necessary to avoid the effects of chronic activation of ARE, which include apoptosis and cell death [34], [35]. In the presence of inflammatory stimulation, where differential protein expression becomes more pronounced, as do elevations in H_2_O_2_, cytokine production is increased. Conversely, increases in Nrf-2 activity decrease production of IL-8 and IL-6. Therefore, chronic activation of Nrf-2 may be repressed at the cost of increased cytokine production.

In addition to increasing H_2_O_2_ levels, decreases in Nrf-2 activity in CF cells decrease TRX-1 and GST expression. These proteins are related to the glutathionylation of NF-κB [36], which occurs after prolonged oxidative stress in normal cells and prevents over-activation. Therefore, decreased Nrf-2 activity would be expected to prolong NF-κB activation, and this is consistent with previous data on CF cell line cytokine production [37]. While non-CF cell lines cease to produce GM-CSF and IL-6 by 6 hr and IL-8 by 24 hr, CF cells continue to accumulate these cytokines for the duration of the experiment (48 hr) [37], suggesting a diminished ability of CF cells to inactivate NF-κB once it is activated. Therefore, Nrf-2 down regulation in CF cells may affect cytokine production both by enhancing activation and reducing inactivation of NF-κB. Interestingly, the disruption of Nrf-2 expression in transgenic mouse models alone is sufficient to result in a proinflammatory phenotype in the lungs [38] and bowels [39] of these animals. The fact that both of these phenotypes are hallmarks of CF pathology lends credibility to the relevance of our findings to the disease in humans.

**Figure 8:**
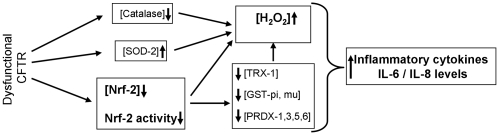
Schematic of the mechanism of H_2_O_2_ regulation its impact on inflammation in CF. Our proteomic analysis of *in vitro* and *in vivo* models of CF indicate differential expression of redox proteins that regulate H_2_O_2_, predicting an increase in peroxide, which we confirmed by biochemical analysis. The mechanism involves a paradoxical decrease in Nrf-2 expression and activity caused by the loss of CFTR function in CF cells. Furthermore, we demonstrate the effect of this phenomenon on inflammation in CF.

In the clinical context, oxidative stress is elevated in the CF airway. Decreases in lumen GSH concentrations [25], and increases in nitrate levels [40] predispose the CF airway to an inability to handle oxidative insult. The drastic influx of inflammatory cells, which produce oxidants to kill bacteria, introduces a heavy oxidative challenge. In this environment diminishment of Nrf-2 activity will exacerbate activation of redox sensitive cascades, including the NF-κB pathway [9], [15], [16]. Excessive production of inflammatory cytokines is a characteristic of the CF lung and is a major contributor to exacerbated pulmonary inflammation that leads to lung failure and morbidity [2]–[8]. Controlling inflammation in CF patients has been shown to be beneficial [2]. Therefore, since activating Nrf-2 controls cytokine production, our findings may have major therapeutic implications. Our data support this notion. Figure 8 schematically demonstrates the redox mediated mechanism of cytokine production in CF epithelia.

The relationship between Nrf-2 activity and control of inflammatory cytokine production make the limited activation of Nrf-2 a good therapeutic target for CF therapy. NAC (increases glutathione), Se (increases TRX, GST, and PRDX activity), tBHQ (stabilizes Nrf-2), and the over expression of Nrf-2 counteract increases in H_2_O_2_ in cells. In CF cells, these treatments decrease the level of IL-6 and IL-8 production to normal levels. Importantly, treatment with increased levels of these antioxidants does not fully inhibit inflammatory responses, which would be deleterious in a CF lung susceptible to infection [2]. Therefore, approaches that control steady state H_2_O_2_ levels, a key contributor to excessive cytokine prodcution, would be expected to normalize inflammatory responses in CF epithelial cells.

## Materials and Methods

### Materials

Cell culture media [37] and materials for proteomic experiments [41] were obtained from sources identified previously. Precast gels and IPTG pI strips are purchased from Biorad laboratories (Hercules, CA). Gelcode Blue™ gel Coomassie stain and sequencing grade trypsin are obtained from Pierce Biotechnology (Rockford, IL). Honeywell Burdick and Jackson solutions for reverse phase liquid chromatography are supplied by Thermo Fisher Scientific (Waltham, MA). IL-6 and IL-8 cytokine production is assayed by ELISA from Luminex Corp. (Austin, TX). Nrf-2 and Nrf-1 driven luciferase expression plasmid is obtained from Panomics, Inc. (Fremont, CA). Tert-butyl hydroquinone (tBHQ) is obtained from Sigma-Aldrich (St. Louis, MO). Luciferase assay reagents are purchased from Promega Corp. (Madison, WI). The Nrf-2 mammalian expression plasmid was a gift from Dr. Mark Hannink. The pCI-neo mammalian expression control plasmid was purchased from Promega Corp. (Madison, WI).

### Cell model systems

We use three airway epithelial cell models; one primary and two immortalized.

### Primary cells

To study well differentiated cells, human tracheal epithelial (HTE) cells are harvested from cadaveric donors and cultured on filters at an air-liquid interface, to produce high-resistance, polarized airway epithelia with cilia and mucus producing cells that are free of macrophages, neutrophils, and bacteria. To establish the CF phenotype we apply CFTR_Inh_-172 [gift from Alan Verkman, 42], which specifically and reversibly inhibits CFTR, but not MDR or other K^+^ or Cl^−^ channels at the concentrations used for our studies (20 µM) [24]. This treatment for 72 hrs also induces some secondary defects observed in CF, including increased Rho A, decreased Smad 3, and increased cytokine responses to PAO1 and TNF-α and IL-1β [24]. The effects of CFTR_Inh_-172 were mediated by the inhibition of CFTR, as it has no effect on cytokine production by the CF phenotype 9HTEo^−^ pCEP-R and 16HBEo^−^ AS cells, where CFTR activity is already absent.

### Immortalized cells

For cell lines, we study the 9HTEo^−^ pCEP and pCEP-R cell pair; and the 16HBEo^−^ sense (S) and antisense (AS) cell pair. Methods for the maintenance of the 9HTEo^−^ and 16HBEo^−^ cell models have been described [37]. Cell pair identity is verified with Western blot analysis for the presence or absence of the R-domain in the 9HTEo^−^ or CFTR in the 16HBEo^−^ cells.

For the 9HTEo^−^ model, cells are stably transfected with pCEP (empty vector) to produce the normal controls, or pCEP-R (vector encoding the regulatory (R) domain of CFTR) to produce cells that lack CFTR function and are functionally CF [37]. Cells are grown in Dulbecco's minimal Eagle's medium (DMEM) supplemented with 10% FBS and 2.5 mM L-glutamine and maintained under selection with 40 µg/ml hygromycin at 37°C in 95% air/5% CO_2_. Cells are grown in T75 flasks and harvested during log phase to avoid contributions of quiescence to alterations in whole cell protein.

For the 16HBEo^−^ model, cells are stably transfected with sense (S) or antisense (AS) constructs of CFTR (nucleotides 1–131). These cells are grown in the same media used for the 9HTEo^−^ cells, but with replacing hygromycin with G418 as the selection agent.

Both the 9HTEo^−^ and 16HBEo^−^ cell line model systems have been extensively studied and shown to exhibit many of the inflammatory and signaling defects observed in the airways of CF patients [37].

### Inflammatory stimulation of the cells and measurement of cytokines

TNF-α and IL-1β were used to stimulate NF-κB mediated inflammatory responses, as previously described [37]. Briefly, TNF-α/IL-1β (10 ng/ml each) are applied to cells, incubated overnight, then processed for experiment. For experiments with additional treatments, such as *N*-acetyl cysteine, reagents are added 2 days previous to or concurrently with TNFα/IL-1β. Following the desired time interval, media from stimulated and non-stimulated cells are harvested and prepared for analysis. An aliquot of media from stimulated cells is assayed by for IL-8 and IL-6 levels by ELISA.

### H_2_O_2_ concentration measurement

For H_2_O_2_ measurement, 25 µl of cell lysate is mixed with 25 µl of working solution of 100 µM Amplex Red reagent and 0.2 U/ml HRP and incubated at RT for 30 mins in the dark. Standards and samples for both assays are analyzed for fluorescence at 544 nm excitation and 590 nm emission.

### SOD activity

5 µl of freeze/thawed cell homogenate in PBS is mixed with 40 µl solution of water-soluble tetrazolium salt (produces a water-soluble formazan dye upon reduction with a superoxide anion). A 0.3 mU/ml xanthine oxidase (XO) solution is added and the solution incubated at 37°C for 20 mins. The production of superoxide by XO produces the formazan dye, which can be detected by measuring absorbance at 450 nm. SOD activity inhibits this reaction and thus the extent of inhibition of this reaction by sample compared to control (dd H_2_O) indicates the level of SOD activity in a given sample.

### Modulating redox state and biochemical outcome measures

We modulate cell redox state in several experiments. All concentrations refer to final concentration in media and reagents are added to media for the duration of the experiment. Control cells are untreated. 1) We add 1, or 10 µM H_2_O_2_ to induce oxidative stress. 2) To alleviate oxidative stress, we add 1, or 10 mM NAC+10 units/ml catalase. 3) We test the effect of selenium supplementation of media (0.1, or 1 µM Se). Se increases the activity of seleno-proteins such as TRX-1, PRDX-1 and PRDX-6. 3) We treat cells with 20 µM CFTR_inh_-172 inhibitor of CFTR [24], [42].

### Cell harvest and homogenate preparation

Cells are harvested during log phase. Stimulated or unstimulated cells are trypsinized, spun down and the cell pellet extensively washed with FBS containing media then PBS to completely inactivate and remove trypsin. Pellets are incubated in 1 ml of cell lysis buffer (0.3% SDS, 200 mM DTT, and 30 mM Tris) at 80–90°C for 5 minutes. Once the homogenate cools, a DNAse/RNAse cocktail is added and the sample is incubated for 10 min. Aliquots of the sample are subjected to Bradford assay to determine protein concentration. Acetone is then added to precipitate protein, the precipitate is pelleted by centrifugation, and the acetone is decanted. The protein is then solubilized and denatured in the appropriate volume of urea buffer (7 M urea, 2 M thiourea, 4% CHAPS, and 50 mM DTT) to achieve a 5 mg/ml protein concentration.

### Two-dimensional gel electrophoresis

First dimension isoelectric focusing (IEF) is performed with 11 cm immobilized pH gradient IPG gel strips with overlapping pI ranges (3–10, 4–7, and 5–8). 200 µl of sample is added to an IPG gel strip in an IEF rehydration tray. The strip is rehydrated for 8–12 hours with the application of 50 V at room temperature, then prepared for SDS-PAGE by incubation in reducing buffer (50 mM Tris, 6 M urea, 100 mM DTT, 30% glycerol, and 2% SDS) for 15 min then in alkylating buffer (50 mM Tris, 6 M urea, 250 mM iodoacetamide, 30% glycerol, and 2% SDS) for 15 min at room temperature. For the second dimension, the IPG strip is embedded in 1% agarose on top of a 12.5% acrylamide precast gel. SDS-PAGE is performed for 1 hour at 200 V. Gels are then fixed, washed, and stained with Coomassie blue.

### Gel map imaging and quantitation

Two dimensional gels of whole cell protein are scanned using a Bio-Rad QS-800 Calibrated Densitometer Gel map imaging and quantitation. Images acquired with a GS-800 densitometer are imported into the PDQuest™ 2-D analysis software and compared. Once gels are manually oriented using a few matched protein bands, the software automatically matches all detected bands on comparative gels, and measures band densities. Five gels for each cell type under each condition are averaged and used in our comparisons.

### Protein digest preparation

Protein hydrolysis is performed in-gel using porcine trypsin as previously described [41]. Briefly, protein bands are excised from the gel using a gel spot cutter, washed with 50% Methanol/5% acetic acid 3 times for 1 hr at room temp., dehydrated with acetonitrile, rehydrated with 100 mM ammonium bicarbonate, dehydrated with acetonitrile, and completely dried in a vacuum centrifuge. Gel pieces are then rehydrated with a 50 mM ammonium bicarbonate containing 20 ng/µl trypsin on ice for 10 min. This solution is then removed and 10 µl of 50 mM ammonium bicarbonate is added to the gel fragment. The digestion is allowed to occur overnight at room temp. Tryptic peptides are extracted from the gel into 30 µl of 50% acetonitrile/5% formic acid, centrifuged under vacuum until acetonitrile is removed and the volume is adjusted to 20 µl of 1% acetic acid analysis by LC MS-MS.

### Protein identification by mass spectrometry

The LC MS-MS system is the Thermo Finnigan LCQ-Deca XP plus ion trap mass spectrometer with a nanospray ion source interfaced to self-packed 10 cm×75 µm Phenomenex Jupiter C18 reversed-phase capillary chromatography columns. Samples are loaded manually. Aliquots (2 µl) of an extracted tryptic digest are injected onto the column and the peptides are eluted by an acetonitrile/0.05 M acetic acid gradient at a flow rate of 0.2 µl/min. The nanospray ion source is operated at 1.8–2.2 kV. The digest is analyzed using the data dependent multitask capability of the instrument acquiring full scan mass spectra to determine peptide molecular weights and product ion spectra to determine amino acid sequence in successive or “tandem” scans. This mode of analysis produces ∼4000 collisionally induced dissociation (CID) spectra of ions ranging in abundance over several orders of magnitude (not all CID spectra are derived from peptides). These data are digitally stored using the Xcalibur™ software. Data analysis is performed using all CID spectra collected in the experiment to search the NCBI non-redundant database with the Mascot and Bioworks search program.

### Measurement of IL-6 and IL-8

We measure the production of the IL-6 and IL-8 pro-inflammatory cytokines by testing media from treated cells by Luminex™ ELISA, as previously reported [43].

### Assays for Nrf-2

We test Nrf-2 activity by measuring firefly luciferase expression driven by the Nrf-2 promoter for glutathione-S-transferase (GST) (sequence: GCT CTT CCG GTG CTC TTC CGG TGC TCT TCC GGT GCT CTT CCG GT). Cultured cells were grown to 80% confluence in 24 well plates. On the day of experiment, growth medium was replaced with transfection medium consisting of Opti-MEM media, 0.9 µg Nrf-2 driven firefly luciferase, and 0.1 µg CMV driven Renilla luciferase (transfection efficiency control). Each sample well was transfected with 1 µg total plasmid DNA complexed to lipofectin, as previously described [44]. Control transfections were carried out under identical conditions with a Nrf-1 driven firefly luciferase expression plasmid. With the exception of the promoter region, this construct is identical to the Nrf-2 driven luciferase vector. After 3 hours transfection media is removed, cells are washed, and fresh growth media is added. Two days following Transfection, cells are lysed, cell debris are centrifuged, and supernatants are assayed by dual luciferase assay. Expression levels are expressed as the ratio of Firefly to Renilla luciferase activity, normalized to non-CF controls. For studies of the effect of the overexpression of Nrf-2, we transfected cells with a Nrf-2 mammalian expression plasmid (pCI vector backbone) that was gifted us by Dr. Mark Hannink. Cells assays are performed 48 hrs. post transfection. The construction of this plasmid has been previously described [45]. Transfection was titrated in CF cells to determine the amount of DNA necessary to produce levels of Nrf-2 expression similar to those observed in normal cells. Transfection with 0.1 µg of plasmid DNA/5×10^5^ cells/well was sufficient to achieve the desired level of Nrf-2 expression. Control transfections were conducted with the pCI-neo vector lacking the Nrf-2 gene (empty vector).

### Animal protocol

All animal protocols were approved by the Case Western Reserve University Institutional Animal Care and Use Committee. We compared 8–12 week old B6.129S6- *Cftr^tm2Mrc^* mice to wild type (WT) littermates. The B6.129S6- *Cftr^tm2Mrc^* mice are R117H murine Cftr mutants that have been back-crossed into the C57BL6j background [46]. Age and sex matched CF and normal littermate mice were euthanized by CO_2_. Nasal tissue that contains nasal epithelia (NE) is excised as previously described [20]. Whole lungs are harvested, washed and perfused with PBS to decrease blood content, and flash frozen for protein processing. Comparison of 2D gels from 5 CF and 5 WT mice were conducted for nasal epithelia and whole lung.

### Statistical analysis

Data are expressed as mean±standard error of the mean (SEM). Significance was tested by ANOVA with repeated measures correction or paired t test [47].

## Supporting Information

Table S1 Parameters for tandem mass spectrometric identification of differentially expressed proteins.
(0.03 MB DOC)

Figure S1 Representative CID spectra used in the MS identification of proteins *in vitro*. Data dependent isolation was used to select abundant ions for MS/MS analysis. Collision induced dissociation (CID) of parent ions shown above produced spectra of b and y fragment ions, the masses of which were used to decipher the sequence identity of each parent ion. Spectra for peptides from human catalase (A), GST-pi (B), PRDX-1 (C), PRDX-2 (D), SOD-2 (E), and TRX-1 (F) are shown.
(0.77 MB TIF)

Figure S2 Total cell superoxide dismutase activity in epithelial cell models. Normal and CF 16HBEo− or 9HTEo− matched cell line pairs were assayed. Unstimulated cells are compared to cells stimulated with TNF-α/IL-1β (10 ng/ml each). * connotes significant difference (p<0.05) from unstimulated normal control, while ** connotes significant difference (p<0.05) from unstimulated and stimulated normal control. Each data bar represents the average of 6 replicate wells in 4 experiments.
(0.26 MB TIF)

Figure S3 Representative CID spectra used for the MS identification of protein *in vivo*. Data dependent isolation of abundant ions for MS/MS analysis was used. Collision induced dissociation of these abundant parent peptide ions produced fragment ions representing the sequence identity for mCatalase (A), mGST-mu (B), mPRDX-3 (C), mPRDX-5 (D), mPRDX-6 (E), and mSOD-2 (F) are shown.
(0.67 MB TIF)
