# Supplementary material for: Dysfunction of Nrf-2 in CF Epithelia Leads to Excess Intracellular H2O2 and Inflammatory Cytokine Production
Source: PLoS One. 2008 Oct 10;3(10):e3367. doi: 10.1371/journal.pone.0003367 (PMC2563038; doi:10.1371/journal.pone.0003367)
Supplement: Table S1 — Parameters for tandem mass spectrometric identification of differentially expressed proteins. (0.03 MB DOC) [file pone.0003367.s001.doc]

Table S1. Parameters for tandem mass spectrometric identification of differentially expressed proteins.

| Protein name | Average sequence coverage (%) | Average number of peptide ions | Protein ID probability (*p* value) |
| --- | --- | --- | --- |
| Catalase | 33 ± 4 | 24 ± 2 | <0.0001 |
| GST-pi | 67 ± 10 | 10 ± 3 | <0.000001 |
| PRDX-1 | 28 ± 5 | 6 ± 1 | <0.001 |
| PRDX-6 | 57 ± 7 | 12 ± 2 | <0.0001 |
| SOD2 | 61 ± 2 | 11 ± 1 | <0.000001 |
| TRX-1 | 52 ± 5 | 4 ± 1 | <0.00001 |
