# Supplementary figures and images for: Dysfunction of Nrf-2 in CF Epithelia Leads to Excess Intracellular H2O2 and Inflammatory Cytokine Production
Source: PLoS One. 2008 Oct 10;3(10):e3367. doi: 10.1371/journal.pone.0003367 (PMC2563038; doi:10.1371/journal.pone.0003367)

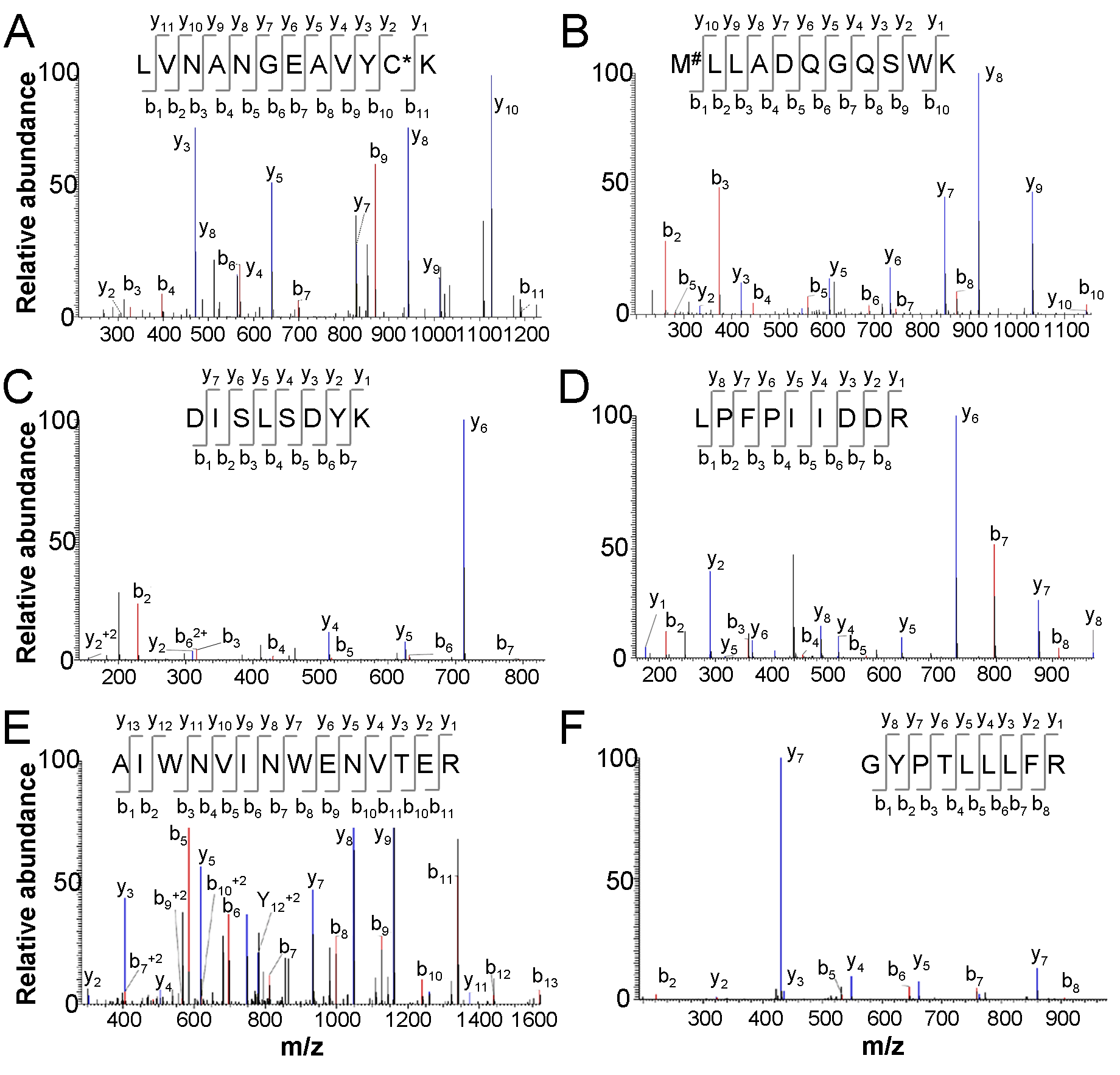

Supplement: Figure S1 — Representative CID spectra used in the MS identification of proteins in vitro. Data dependent isolation was used to select abundant ions for MS/MS analysis. Collision induced dissociation (CID) of parent ions shown above produced spectra of b and y fragment ions, the masses of which were used to decipher the sequence identity of each parent ion. Spectra for peptides from human catalase (A), GST-pi (B), PRDX-1 (C), PRDX-2 (D), SOD-2 (E), and TRX-1 (F) are shown. (0.77 MB TIF) [file pone.0003367.s002.tif]

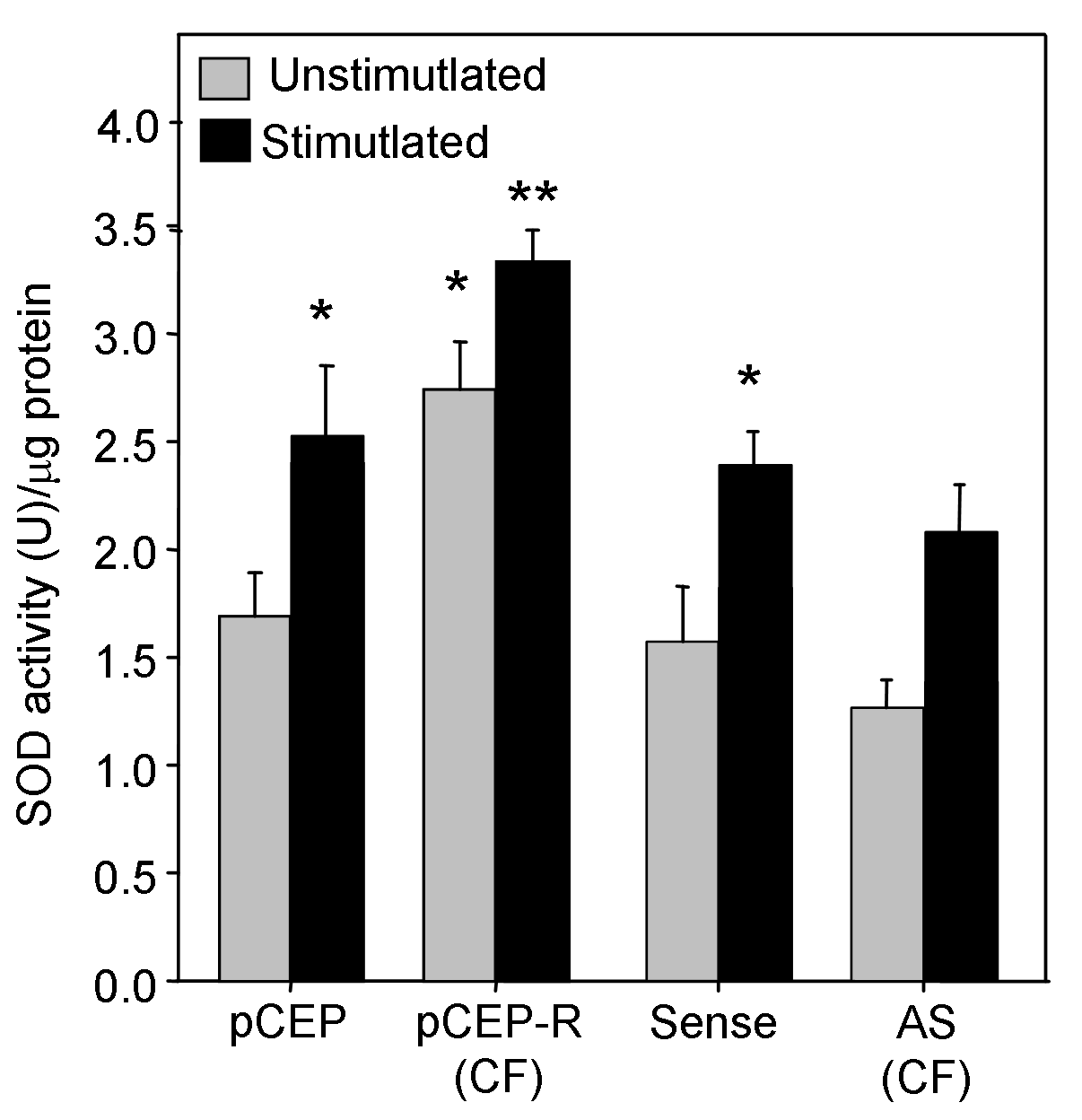

Supplement: Figure S2 — Total cell superoxide dismutase activity in epithelial cell models. Normal and CF 16HBEo− or 9HTEo− matched cell line pairs were assayed. Unstimulated cells are compared to cells stimulated with TNF-α/IL-1β (10 ng/ml each). * connotes significant difference (p<0.05) from unstimulated normal control, while ** connotes significant difference (p<0.05) from unstimulated and stimulated normal control. Each data bar represents the average of 6 replicate wells in 4 experiments. (0.26 MB TIF) [file pone.0003367.s003.tif]

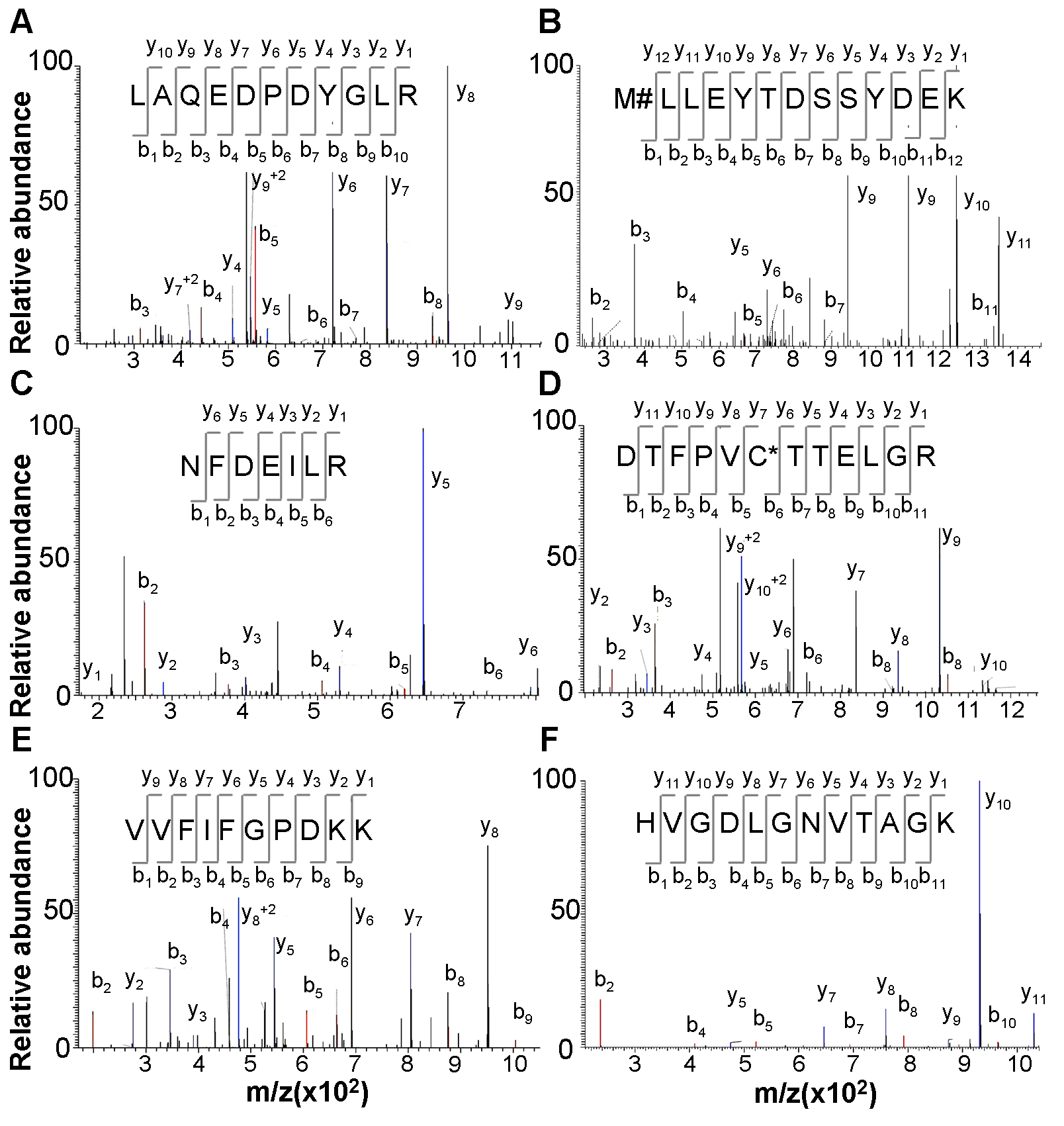

Supplement: Figure S3 — Representative CID spectra used for the MS identification of protein in vivo. Data dependent isolation of abundant ions for MS/MS analysis was used. Collision induced dissociation of these abundant parent peptide ions produced fragment ions representing the sequence identity for mCatalase (A), mGST-mu (B), mPRDX-3 (C), mPRDX-5 (D), mPRDX-6 (E), and mSOD-2 (F) are shown. (0.67 MB TIF) [file pone.0003367.s004.tif]
